# DOLARS, a Distributed On-Line Activity Recognition System by Means of Heterogeneous Sensors in Real-Life Deployments—A Case Study in the Smart Lab of The University of Almería

**DOI:** 10.3390/s21020405

**Published:** 2021-01-08

**Authors:** Marcos Lupión, Javier Medina-Quero, Juan F. Sanjuan, Pilar M. Ortigosa

**Affiliations:** 1 Department of Informatics, University of Almería, CeIA3, 04120 Almería, Spain; marcoslupion@ual.es (M.L.); jsanjuan@ual.es (J.F.S.); 2 Department of Computer Science, Campus Las Lagunillas, 23071 Jaén, Spain; jmquero@ujaen.es

**Keywords:** activity recognition, real-time, smart home, sliding windows, distributed system

## Abstract

Activity Recognition (AR) is an active research topic focused on detecting human actions and behaviours in smart environments. In this work, we present the on-line activity recognition platform DOLARS (Distributed On-line Activity Recognition System) where data from heterogeneous sensors are evaluated in real time, including binary, wearable and location sensors. Different descriptors and metrics from the heterogeneous sensor data are integrated in a common feature vector whose extraction is developed by a sliding window approach under real-time conditions. DOLARS provides a distributed architecture where: (i) stages for processing data in AR are deployed in distributed nodes, (ii) temporal cache modules compute metrics which aggregate sensor data for computing feature vectors in an efficient way; (iii) publish-subscribe models are integrated both to spread data from sensors and orchestrate the nodes (communication and replication) for computing AR and (iv) machine learning algorithms are used to classify and recognize the activities. A successful case study of daily activities recognition developed in the Smart Lab of The University of Almería (UAL) is presented in this paper. Results present an encouraging performance in recognition of sequences of activities and show the need for distributed architectures to achieve real time recognition.

## 1. Introduction

Smart environments allow the monitoring of human activity of inhabitants in an increasingly less invasive way [1]. Activity Recognition (AR) has been among the main topics of investigation in relation to the development of smart environments [2,3]. Concretely, ambient-assisted living has been adopted in several contexts, such as, smart homes and healthcare applications [4] aiming both at improving the quality of care services and allowing people to stay independent in their own homes for as long as possible [5].

AR aims to develop predictive models which detect human action and their goals [6] within a smart environment. They aim to provide assistance to the inhabitant by, for instance, detecting emergency situations based on the data being observed [7]. In the initial work in the field of AR, binary ambient sensors [8] (such as passive infrarred sensors or open door) dominated the sensing of human behaviours being proposed as suitable devices for describing daily human activities. Nowadays, a new generation of devices has highlighted the capability of sensing the activity from a closer point of view of the user [9]. Between them, we highlight location [10] and wearable [11] devices. So, in the context of smart environments, the new generation of non-invasive devices is being combined with the use of ambient sensors. New methodologies have been necessary to provide a fusion of spatial and temporal features [12] to relate heterogeneous sensors with AR. Moreover, in real-time requirements for AR, the on-line evaluation of sensor data has been faced using multi-feature sliding windowing approaches [13,14].

In addition, in the context of ambient-assisted living and AR, the concept of smart homes/environments emerged as a prolific research topic which has enabled the development of the Smart Labs [15] in scientific and research centers. The design, starting and configuration of a Smart Lab from scratch is an arduous task which involves device selection, data collection together with the synchronization and orchestration of remote services in a real-time deployment [16]. The current approaches of AR fall into recent paradigms such as Edge Computing [17] or Fog Computing [18] which edge the data processing and services close to where data are collected.

Based on the background of the current state of technology, in this work we present the on-line activity recognition platform DOLARS (Distributed On-line Activity Recognition System) where data from heterogeneous sensors are evaluated in real time in a distributed architecture, including binary and new generation of wearable and location sensors.

DOLARS integrates a distributed architecture where: (i) stages for processing data in AR are deployed in distributed nodes, (ii) temporal cache modules compute metrics which aggregate sensor data for computing feature vectors in an efficient way, and (iii) publish-subscribe models are integrated both to spread data from sensors and orchestrate the nodes (communication and replication) for computing AR. The system which has been deployed in the Smart Lab of The University of Almería (UAL) is presented in this paper together with a description of the space and component which configure it. Lastly, a case study of high rate AR under real-time conditions has been developed in the context of daily activities.

In the remainder of this article, we delve into the description of the proposed approach with the contents organized as follows—Section 2 reviews related works and state of the art of similar approaches. Section 3 presents the proposed methodology. Section 4 introduces the evaluation of the methodology analyzed in a real-world dataset collected in the Smart Lab of The University of Almería (UAL). Finally, in Section 5, conclusions, ongoing and future works are discussed.

## 2. Related Works

In this section, we provide a summary of key works on the field of Activity Recognition, Smart Environments and sensor collection.

As we detailed previously, nowadays integrating heterogeneous sensors which describe the interaction between objects, movements and location is suggested to enable a high performance in AR [19] and Ambient Assisted Living (AAL) [20]. From the initial binary sensors, which have been proposed from initial works [21] to recent literature [22,23], the next generation of sensor based on wearable devices has included inertial sensors in activity recognition approaches using machine learning models [11] and deep learning [24]. The use of inertial sensors in mobile and wearable devices has provided an open research field which is able to collect data in a naturalistic way in both indoor and outdoor smart environments [25]. These devices coexist with traditional binary sensors to enable rich AR by means of sensor fusion [26].

Recently, indoor location devices have been integrated to include the position and tracking of the user within smart environments. The use of location information improves the AR [11] regarding isolation information from wearable devices. In the context of location sensors, Ultra Wideband (UWB) is allowing to reach extremely high accuracy in indoor contexts [27]. The capabilities of UWB have even enabled the multi occupancy with high accuracy in real-time environments [28].

The use of heterogeneous devices require a fusion layer where different descriptors and metrics from sensor data, which are based on the nature of the information and devices which collect data, are integrated in a common feature vector [12]. However, the features and window size for evaluating binary sensors [19] or wearable sensors [29] differ. For that purpose, it is necessary to provide ad hoc processing for each type of sensor data [30]. Automatic extraction of features, such as Convolutional Neural Networks, present similar or slightly lower results in daily activity datasets [31]. Moreover, the use of multiple temporal windows have provided encouraging results [19,23,32] because relevant information is captured in different segments and reduce the impact in the decision making of selecting window sizes [33].

In on-line activity recognition, the feature extraction is developed by sliding windows [34] which compute the features from current and past sensor data evaluating the AR in real time for each time step [23]. The increase of performance by delaying the decision making of the developed activity when including preceding and ongoing sensor data in on-line AR is noticeable [35].

At the same time, the collection of sensors which enable learning models to predict human activities have encouraged the development of smart environments, where evaluation of activity recognition approaches are developed. Between the wide range of smart labs and smart homes described in the literature, we highlight:

- MIT Smart House, better known as PlaceLab [36], which is considered the first Living Lab conceptualized as such [37]. It includes a location-enhanced mobile computing system using radio beacon [38].
- Ubiquitous Home which integrates a robot and floor and ambient sensors [39].
- Welfare Techno Houses [40] presented an automated monitoring system with toilet, bed and bathroom sensing.
- Pervasive Computing Lab from Ulster University integrates assisting technologies for smart homes, independent living, and healthcare monitoring and diagnosis [15].
- HomeLab which consists of a large number of geographically distributed households with a common framework [41].
- Iowa State University Smart Lab [42], where a flexible software based on service oriented architecture enables us to discover and access sensor data in real time.
- Cloud-assisted Agent-based Smart home Environment where a framework for the design and implementation of smart home applications focused on activity recognition in home environments is proposed [43].
- SPHERE in a Box is a practical and scalable activity monitoring Smart Home Kit. Its design consists of a low cost and consumption wearable that only has an accelerometer and a Bluetooth Low Energy (BLE) connection along with 4 gateways [44].
- UJAMI SmartLab is a real apartment with multiple and heterogeneous sensors and actuators that are connected by a unified middleware [16].

However, distributed architectures for smart homes are necessary to face the fact that the volume, velocity and variation of such sensor data, its storage and retrieval has become a big data problem [45]. Firstly, assisting agents have been proposed to personalized assistance in terms of the needs of the user by recognized activities [46]. Agents have been integrated with Ontologies to interpret data and reason in context aware domains, which enhances the capabilities of data interpretation and inference. Agent integrated in middleware services enhance the dynamism inherent in Ambient Intelligence environment [47], where Jadex was integrated to support agent modeling and reasoning engine. Secondly, streaming or online within activity recognition opened new challenges and requirements for architectures in terms of segmentation [34]. In Reference [48], an architecture is proposed to support the stages in the activity recognition (pre-processing, feature extraction, and feature selection tasks) which are distributed in modules to provide machine learning methods dynamically to the framework. Fuzzy logic has provided encouraging results in handling heterogeneity and uncertainty of sensor processing. In Reference [49], an activity recognition architecture based on fuzzy logic was proposed, where multiple nodes collaborate to produce a reliable recognition result from unreliable sensor data. In Reference [50], a fuzzy linguistic representation of mobile and ambient sensors was integrated to provide a drastic reduction of the communication burden based on publication-subscription paradigm [50].

More recently, fog/edge-inspired distributed architectures have been proposed to process data and collaborative services which are embedded within smart objects to cooperate between each other to reach common goals [51,52]. For example, in Reference [53], authors collect, analyze and identify ambient events that represent abnormal behavior or dangerous health conditions in large-scale scenarios. In addition, some other systems, such as DCR described in Reference [54], are composed by agents who observe sensor data, make predictions, communicate amongst each other and collaborate in order to identify user activities, without having to manage all these operations in centralized cloud systems.

Based on the related works described in this section, in upcoming sections we detail a further architecture and methodology to develop on-line AR from real time processing of heterogeneous sensors which include location sensors, inertial wearable devices and ambient sensors deployed in the new Smart Lab of UAL in Spain.

## 3. Materials and Methods

This section describes the infrastructure where the experiments have been carried out, as well as the different devices and protocol architecture used to carry out the work. It has been configured into four different subsections. Therefore, Section 3.1 describes the distribution and infrastructure of the SmartLab from UAL. Section 3.2 makes a brief description of additional devices that have been deployed at the smart-home and also lists the daily activities that have been selected to their recognition. Section 3.3.2 analyzes the spatial temporal features of different sensors, and finally Section 3.3 shows the whole deployed architecture and the protocols that have been considered.

### 3.1. SmartLab from UAL

**Figure 1:**
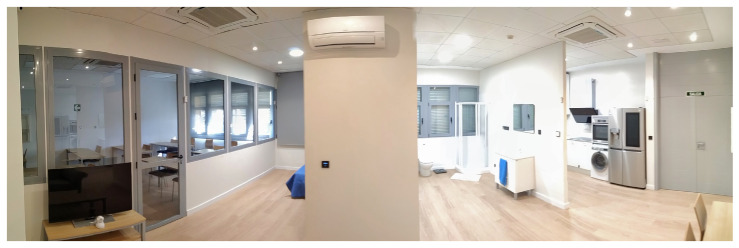
Smart Home of University of Almería (UAL).

At UAL, there is wide range of research groups working at The Computer Science department. The Smart Lab of UAL (Figure 1) is focused on attracting that applied knowledge to develop a space with an innovative technology and artificial intelligence methods to deploy assisting technologies that improve the user’s living conditions. The main interest of developing the smart home was to provide it with Ambient Intelligence (AmI) [55] so that the home can be sensitive, adaptive, and responsive to human needs, habits, gestures, and emotions. One of the main research lines opened by the Smart Lab is focused on Ambient Assisted Living (AAL) and controlling the habits and health of the elderly people, or people with disabilities.

**Figure 2:**
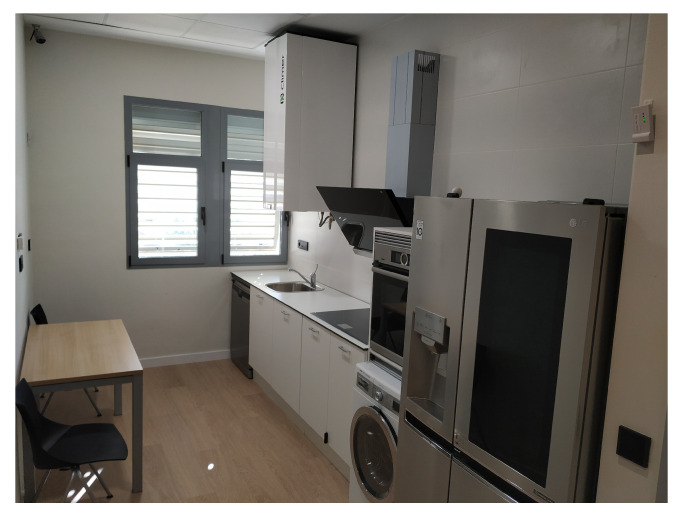
Kitchen.

**Figure 3:**
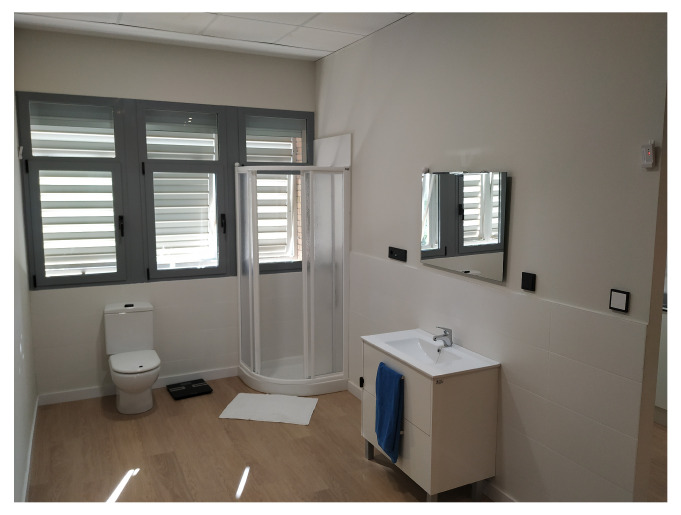
Bathroom.

**Figure 4:**
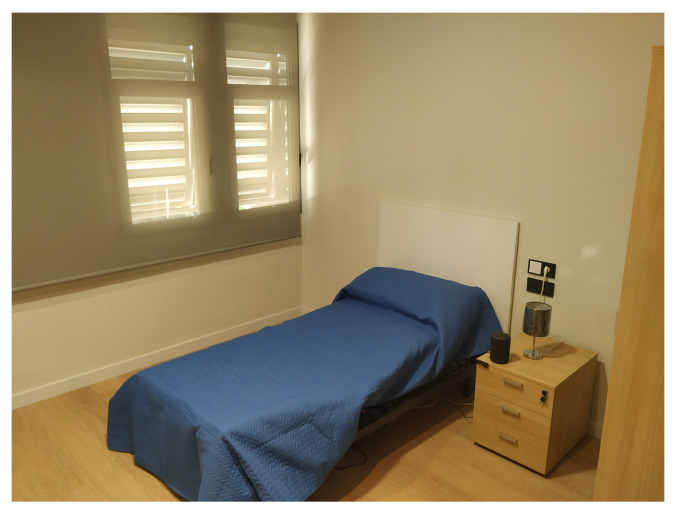
Bedroom.

**Figure 5:**
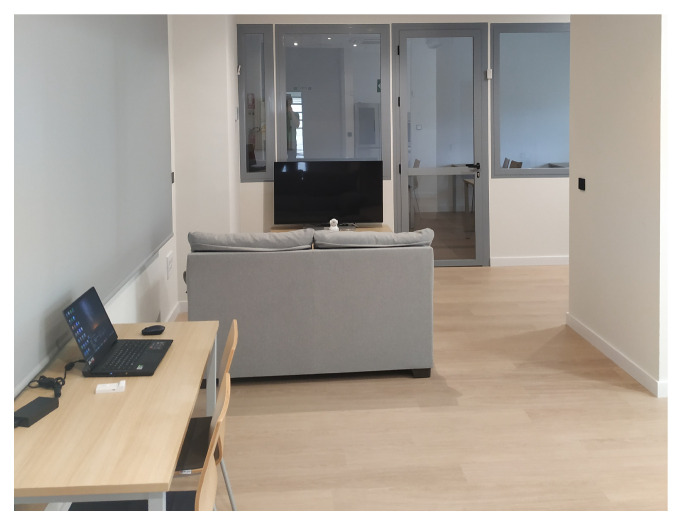
Hall & Living room.

To enable the Smart Lab, UAL has built a diaphanous flat, where open rooms allow accessibility to people with mobility problems. The Smart Home distribution is a kitchen (Figure 2), a bathroom (Figure 3), a bedroom (Figure 4), a hall, and a living room (Figure 5). The laboratory also has an articulated bed for people who are ill or have reduced mobility.

It also includes an observation room where students can interact with the house through their computer equipment as well as observe the reactions and living actions of the inhabitants for a wide range of potential experiments such as technical or human ones.

**Figure 6:**
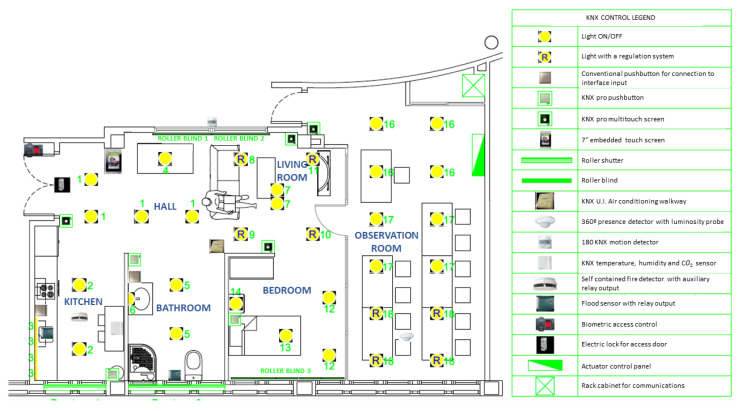
Initial distribution plant of Smart Home of UAL.

For its configuration and simulation of a domotic environment, the smart lab has implemented several types of KNX compatible intelligent devices. KNX is an OSI-based network communications protocol standard for intelligent buildings. The devices are shown in Figure 6. These devices are: Lights (normal and dimmed); push buttons (normal and touch); blinds; roller blinds; air conditioning; presence and motion detectors; temperature, humidity, CO_2_ and flooding sensors; fire detectors; biometric access control and electric lock; built-in touch screen.

The laboratory additionally consists of the following non-KNX protocols and devices: (i) IP security cameras; (ii) HomeConnect devices for Siemens brand dishwasher, and for the Bosch brand oven, hood, glass-ceramic cooker and washing machine; (iii) Smart ThinQ for the fridge.

In addition to the connection of high quality commercial devices, there is a wireless node that allows any other device to be integrated through TCP/HTTP connectivity by WiFI or ethernet, such as a smart TV.

### 3.2. Description of Devices to Sense Daily Activities

The Smart home at UAL has, therefore, a wide variety of sensors and actuators capable of performing certain actions automatically without user intervention with the aim of improving the user experience in the home.

**Figure 7:**
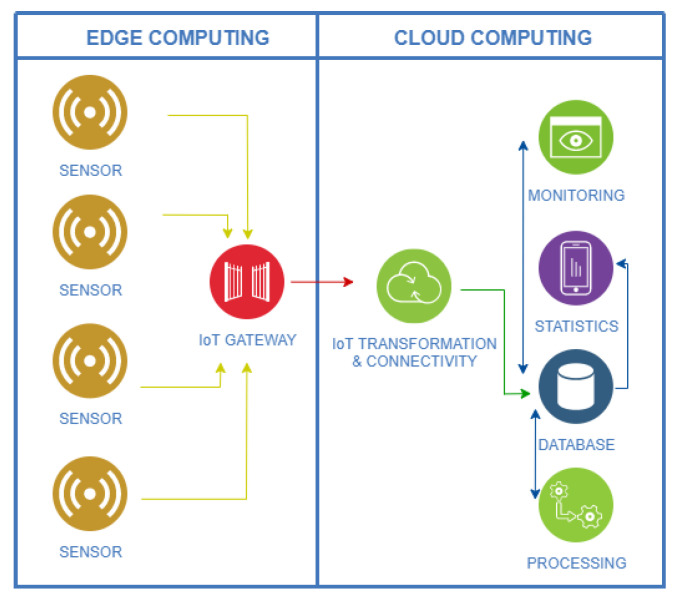
Infrastructure of the Internet of Things (IoT) system.

However, the devices previously described include domotic capabilities in the smart home, but they are not enough to be able to provide data related to the activity of the user. For this reason, new sensors and devices have been deployed in the smart home. Thus, a low-cost and non-invasive IoT platform (Figure 7) based on ambient and wearable devices has been integrated [56]. To implement the system, non-intrusive sensors are deployed in the environment, with the aim of preserving the user’s privacy [57]. Its main objective is to collect the data provided by the inhabitant when interacting with the environment and to analyze them to detect the activities carried out by him/her. In addition, a web tool in the cloud allows the researcher to subscribe the collected data by means of subscription services based on MQTT (Message Queue Telemetry Transport). The web tool mainly allows the researchers to monitor the state of the home in real time and the activities done by the participants. Among other functions, it also provides a view of historical sensor data and lets the researchers manage the situation of sensors in the rooms. In the future, this system will be deployed in real scenarios such as care homes for the elderly, where the web tool will be accessible to caregivers, family and medical staff.

The sensors deployed in the smart-home to collect data to be processed for activity recognition can be classified into three main groups: passive sensors, wearable sensors and location sensors.

Firstly, the passive sensors were incorporated in the Smart Home. The main common characteristic of the passive sensors at the Smart Home is that their communications to the controller(Raspberry Pi) have been implemented by using Z-Wave and they stay in a static way in the home.

Fibaro Motion Sensor is a Z-Wave Plus compatible multi-sensor device, which incorporates a presence sensor, temperature sensor, light sensor and accelerometer. This sensor is mainly used to detect the presence of a user in a room. In future versions of the system, temperature and light sensors will be considered in order to increase detection accuracy.

In the same way, Fibaro Door/Window Sensor 2 is a wireless, battery powered, Z-Wave Plus compatible magnetic contact sensor. Changing the device’s status will automatically send a signal to the Z-Wave controller and associated devices. This sensor allows us to know the state of an object or element in the house that can be open/closed, such as a door, a drawer or a window.

**Figure 8:**
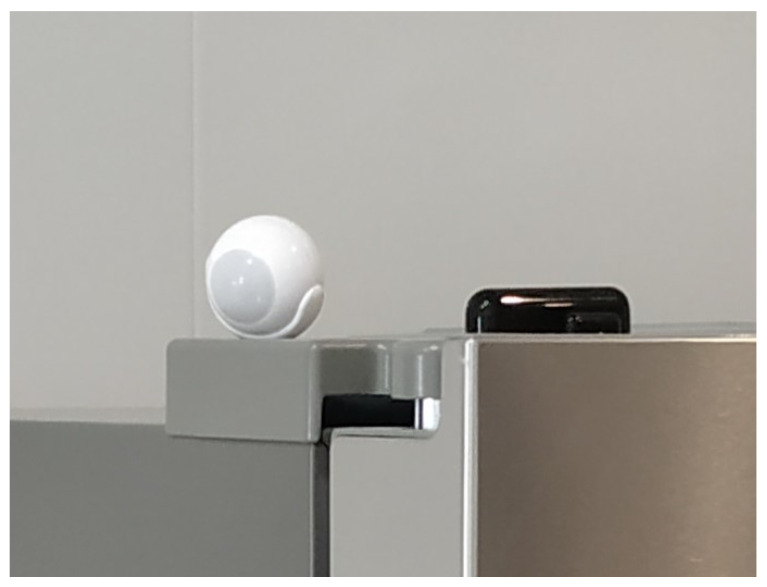
Fibaro Motion Sensor & Door/Window Sensor 2.

These sensors are shown in Figure 8. In this image, they are located on the fridge. The motion sensor activates when a person enters the kitchen and the door/window sensor let us know the state of the freezer.

**Figure 9:**
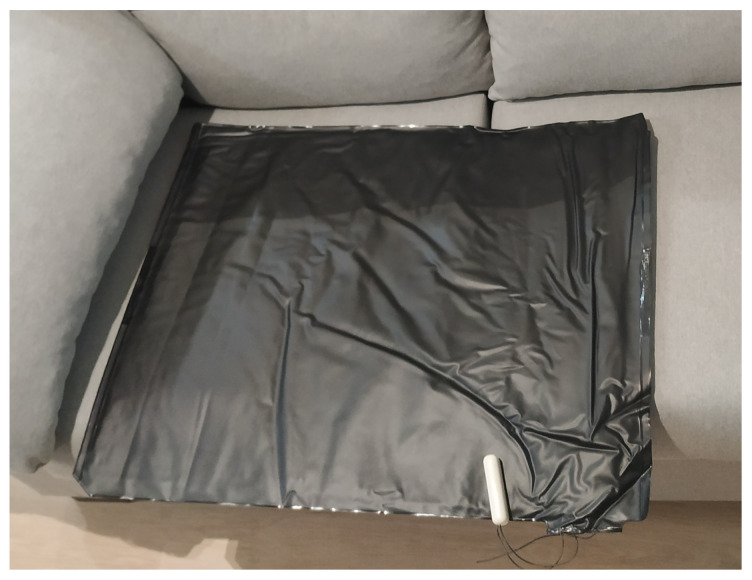
Arun PM3 & Door/Window Sensor.

**Figure 10:**
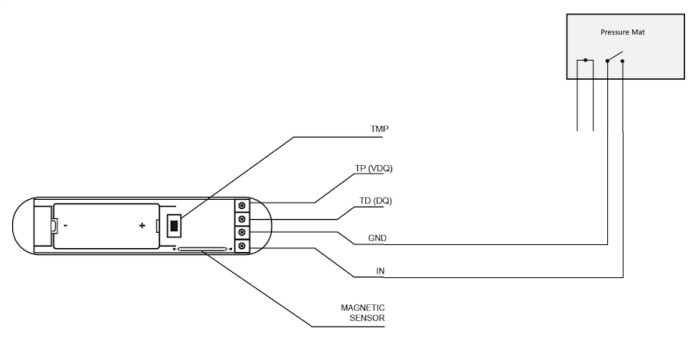
Bridge between Door/Window Sensor and Arun PM3.

In addition, Arun PM3 is a binary pressure sensor, in charge of detecting pressure on its surface. This pressure sensor has been used to check when a person sits on a certain surface. Since the connection of this sensor is made through cables, to communicate with the Z-Wave it is necessary to connect by cable the binary pressure sensor to other Z-Wave compatible devices, for example, Door/Window Sensor. In Figure 9 and Figure 10 the connection between the pressure and binary sensor is shown. In the Smart Home, these sensors are placed under the sofa. As can be seen in the Figure 10, the cable connection between the two devices is almost invisible to the inhabitant.

**Figure 11:**
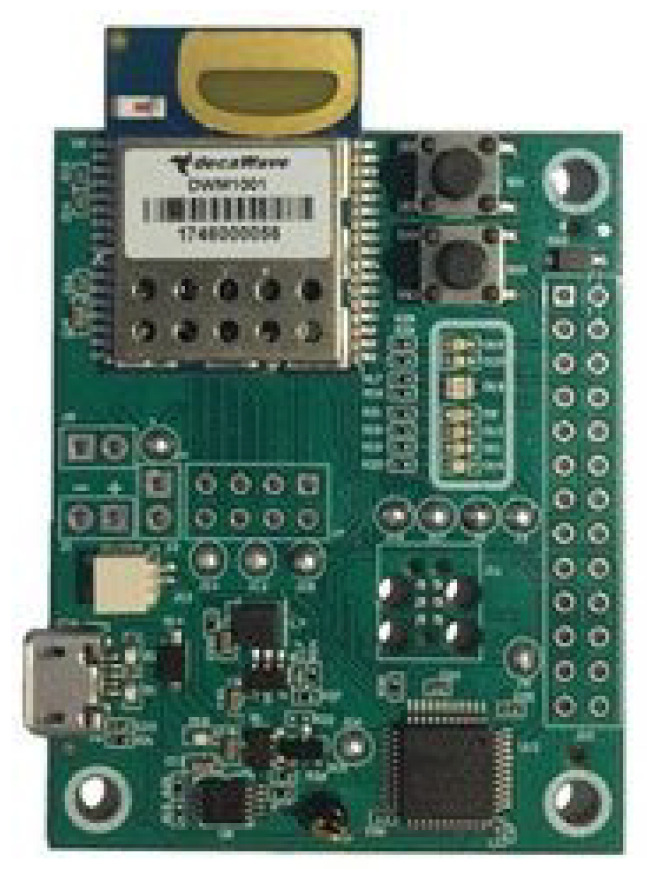
DWM1001 device.

**Figure 12:**
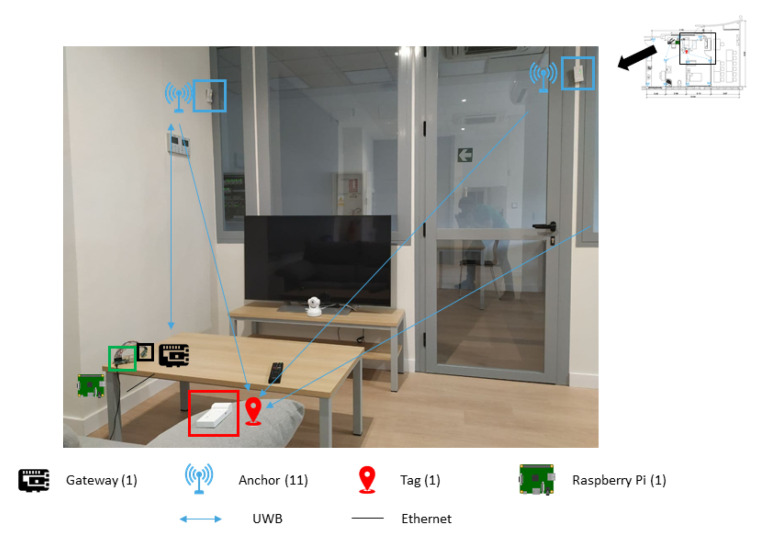
Roles in a Real Time Location System (RTLS) network.

Secondly, as for the location sensors, an RTLS system (Real Time Location System) is installed in the Smart Home. In this case, its communication protocol is UWB (Ultra-Width-Band). Specifically, it incorporates the MDEK1001 Development Kit from the company Decawave. The RTLS network consists of a set of DWM1001 devices (Figure 11), which can have different roles. One device (*Gateway*) is in charge of connecting to a controller element (Raspberry Pi in this case) and acting as a bridge between the RTLS network and the rest of the IoT system. Other devices are incorporated into the house such as *Anchors* being fixed in the laboratory to delimit the surface on which the user is monitored. Additionally, other non fixed DWM1001 devices (*Tags*) work as wearable devices that are carried by the user. In this way they allow monitoring their location with respect to the *Anchors* with a maximal error of 15 cm. Figure 12 shows the structure of an RTLS network with the devices assuming different roles.

**Figure 13:**
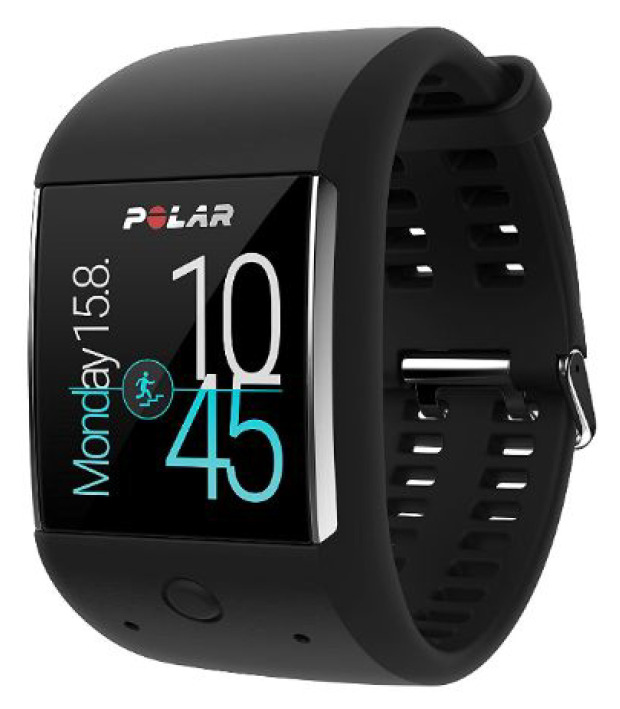
Polar M600 wearable device.

Thirdly, as a wearable device deployed for obtaining acceleration and gyro data from the user when performing certain activities, a smartwatch has been selected (Figure 13). This device is Polar M600, which has been chosen because of its high popularity in relation to the price-quality ratio and also because of the incorporation of acceleration and gyro sensors and the Android Wear OS, thanks to which it is possible to include our own applications which access these data provided by the sensors.

**Figure 14:**
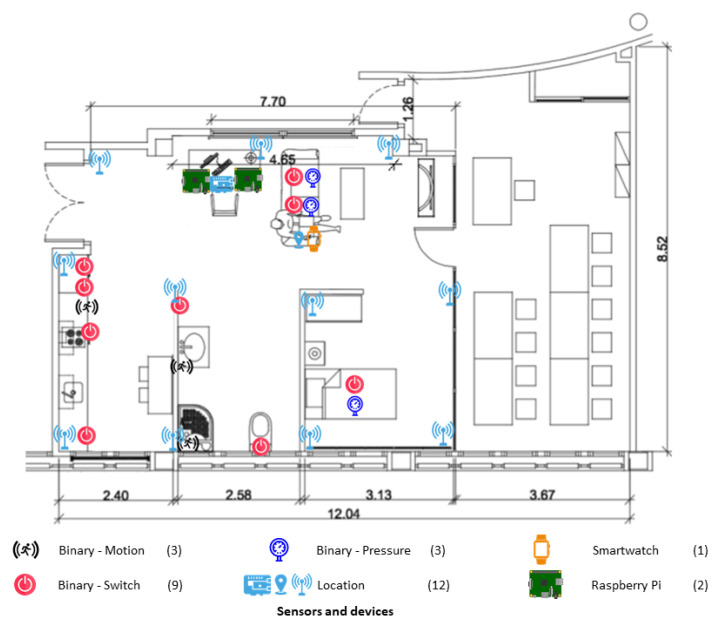
Deployment of the different sensors in the Smart Home.

Finally, Figure 14 shows the deployment of the different types of sensors in the Smart Home.

In Table 1 the total price of the system (in euros) is detailed.

With regard to the activities that have been included in the system, the authors have selected a group of representative activities that we normally do in everyday life (Activities of Daily Living-ADLs). These activities can determine the degree of independence that a person can achieve at home [3]. Also, in Reference [58], some ADL that are found in clinical questionnaires were considered, so we have selected some of them to be included in our work. In addition, other papers such as References [12,59] studied preferential activities to introduce in their systems. In these, the Smart Lab includes similar sensors and devices to ours, so the activities detected can also be detected with systems such as DOLARS. Also, some new activities such as “Using the computer” or “Looking at the smartphone” have been added to our system in order to detect a sedentary lifestyle in adults. Thus, the activities included in the system are:

1. Walking (Walk)
2. Looking at the mobile phone (Smartphone)
3. Sleeping (Sleep)
4. Using the computer (PC)
5. Getting dressed (Dress)
6. Going to the toilet (WC)
7. Combing/Brushing hair (Comb)
8. Brushing the teeth (Brush teeth)
9. Washing the hands (Wash hands)
10. Having shower (Shower)
11. Drinking water (Drink water)
12. Eating (Eat)
13. Sweeping (Sweep)
14. Watching TV (Watch TV)

These activities can be indicators of a person’s status at home. Thus, in the case of elderly people, it may be indicative that the person has some ailment or injury when, for example, he/she stops sweeping, takes longer than normal to shower or stops eating. It is also possible to control the sedentary life of the inhabitants of the dwelling by detecting when a person spends most of his or her time sleeping, with a mobile phone or with a computer. Similarly, it is possible to control the diet of the inhabitants of the home by detecting the number of times they eat, drink water and go to the bathroom throughout the day. Consequently, the activities that have been included allow a study of the life of people living in the smart home and detecting possible anomalies in them to correct them and improve the user’s life.

### 3.3. Architecture for On-Line Activity Recognition from Heterogeneous Sensors

**Figure 15:**
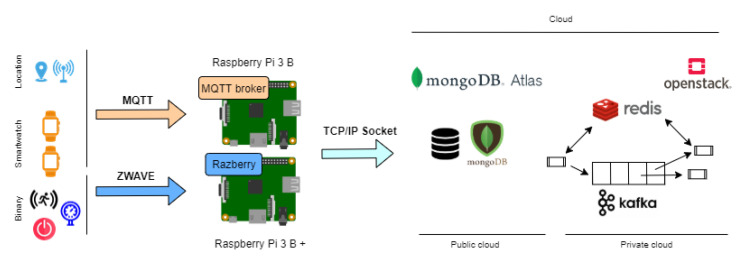
Infrastructure of the system.

In this section, a distributed architecture deployed in the Smart Lab of UAL is presented (Figure 15). It is focused on the scalability with distribution of remote tasks within real-time response which provide on-line activity recognition from heterogeneous sensors.

First, a light publish-subscribe model (MQTT) is detailed to integrate the data from different sensors in a unified way. Second, metrics and temporal features to describe and fuse the information from sensors is described. Real-time computing of temporal features is optimized by a distributed node and cache databases (Redis). At the end, on-line activity recognition is developed by means of real-time distributed services under a reliable publish-subscribe model (Kafka).

#### 3.3.1. MQTT As an Homogeneous Protocol to Integrate Heterogeneous Devices

The on-line activity recognition system incorporated in the house makes use of a large amount of data which come from several devices which are located in the smart home that sense the behaviour of the user. The orchestration, distribution and design of services and modules for developing a scalable architecture for on-line activity recognition is required.

First, in this work, MQTT (Message Queue Telemetry Transport) protocol has been mostly adopted as protocol for integrating information from heterogeneous sensors. MQTT is an Open Source communication protocol in charge of message publication/subscription. This protocol has been adopted because it allows its possible integration into various technologies such as programming languages (including libraries), sensors and controller devices that interact with these sensors. Thanks to this protocol, we establish a permanent connection among devices and data flows from one to another when they are generated.

The role of MQTT as a homogeneous layer is key because mainly IoT devices are connected in heterogeneous protocols. First, several binary sensors, such as pressure, motion and door/window sensors, communicate with the controller by using ZWave protocol. This is a wireless communication protocol mainly used in domotics due to its low battery consumption. Here, a fog node is deployed as gateway between binary sensor and MQTT, receveing data from Z-Wave and translating the information in published messages in MQTT in real time. The access to the ZWave network is secured by the ZWave communication protocol. Some credentials established by the administrator are required each time the user wants to modify or view the network or access the generated data through the API.

Second, the location devices get the location of the user in real time by using the UWB protocol so as to communicate between them. Once the distance is calculated, a fog node is configured as a gateway to enable the translation of this information to MQTT in real time. Finally, the wearable wrist band incorporates an application that collects the data from the accelerometer and gyroscope sensor in real time and sends it to the controller by using the MQTT protocol. In the MQTT communications, a pair of encryption keys have been incorporated to secure the connection between the devices.

The left panel of Figure 15 shows all the devices included and the communication protocols between each sensor and the controller. Next, a subscription fog node receives data from the sensors and devices in MQTT. It checks if the data are correct and stores them in the cloud in order to be processed. In particular, these data are stored in a MongoDB database deployed by the service MongoDB Atlas. This database has been chosen due to its flexibility and scalability. Once the data of the sensor are stored in the database, scripts deployed in several virtual machines process the data in order to recognize the activities which are being carried out by the inhabitants.

The controller of our system (Raspberry Pi) communicates with the cloud server by using wifi protocol.

#### 3.3.2. Combining Spatio-Temporal Features from Heterogeneous Sensor

As defined in the previous section, three types of sensors are included in the smart lab which sense the space and the inhabitant with different data and frequency. First, the binary sensors, which are located in the lab in the ambient space in a static way, generate new data each time the user interacts with them, so the frequency of data generated cannot be predicted. However, this interaction in most cases is not higher than 10 interactions per minute. Second, the configured UWB location sensors produce one notification per second so, in this case, it is enough frequency to detect the position of the user in the smart lab. Third, sensors included in the smartwatch (accelerometer and gyroscope sensors) produce several new notifications per second. The exact number of notifications can be established in the smartwatch by the user. In this work, the frequency has been set to 100 notifications per second from the accelerometer sensor and from the gyroscope sensor. Thus, the device does not send new data every 10 milliseconds, but rather sends a batch of 100 data points every second, enabling lower battery consumption. This configuration offers almost 6 h of continuous monitoring for the data sensor. In this way, this frequency allows movements from people to be detected and therefore some short activities can be recognized.

Taking into account these different notification frequencies from the sensors, in this work a sliding window approach [34] has been adopted. The sizes of these windows depend mainly on the nature of the sensor [19,29]. In this work, each sensor is related to different windows which have been defined in order to consider present and past notifications [12]. Therefore, all sensors have a short term window which represents the current state of the sensor and other middle term windows which represent past notifications. The configuration of multiple windows from current time to past facilitate the parameter impact of the window size as a critical point in activity recognition [12,23]. So, the different window sizes which have been defined for each sensor are shown in Table 2.

In fact, in relation to the binary sensors, two windows have been defined so that the present window, which defines the current state of the sensor, lasts 5 s and the past window lasts 30 s. In relation to second group of sensors, UWB location sensors, which have higher notification frequency than binary ones, their window sizes are smaller. For these sensors, three different windows have been defined. The present or current window lasts only 1.5 s and two past window sizes have also been considered as shown in Table 2. Finally, accelerometer and gyroscope sensors, with the highest notification frequency (100 Hz) require smaller window sizes, in such a way that the current window, which determines a single user movement, lasts only 0.5 s. Some other past windows have to be considered in order to include the current movement into a determinate activity. These past windows have different sizes with a maximal duration of 5 s. Some bigger windows are not included as they could provide useless data and deteriorate other important data gathered.

Once all the sliding windows for each type of sensor have been defined, it is necessary to process the data and generate some important features which will be the input of the developed algorithm. In this work the minimal window size for the feature vector has been fixed to 0.5 s. This minimal window is defined as time-step and indicates that every 0.5 s the system has to provide a recognized activity based on the feature vector [23,33].

In addition, there are several approaches which concern the most important features. In some cases, some basic metrics like maximum, minimum, average and standard deviation are applied to each window [60]. In this work, after making some preliminary experiments with different metrics, the minimum, maximum and average have provided encouraging results, not being necessary the use of other metrics.

Therefore, for the inertial sensors, three characteristic values (maximum, minimum and average) are associated with each window, and depending on the number of windows, a different number of characteristics will be required. In particular, each window of the inertial sensors (accelerometer, gyroscope and location) contains the maximum, minimum and average value of each coordinate. As they have 3 coordinates (x, y and z), each window contains 9 features. Considering that the accelerometer and gyroscope have 4 windows of time, a total of 36 features will be required for each one. The location sensor has 3 windows so it will provide 27 features. On the other hand, in the case of binary sensors, the approach is different because only the average feature has been representative. Therefore, each sensor provides as many features as windows have been defined for the binary sensors. A total of 117 features are computed given the defined windows.

#### 3.3.3. Optimizing the Real-Time Computing of Temporal Features in Cache Databases

In this work, there are several sources which produce data that have to be processed in order to obtain an activity. The challenge of our approach is recognizing user activity in real time in a high frequency defined by a time-step (each 500 milliseconds). Consequently, the communication between the devices and the group of controllers must be constant and simultaneous to the reception of the data from the sensors. As each source (or sensor) has a different frequency and delays and there is a huge amount of information gathered per second. Considering that our system has two low-cost controllers (Raspberry 3B+) it would be quite complicated to design an efficient edge system that supports a kafka broker with several groups of consumers and that simultaneously obtains sensor data, calculates metrics, processes the data, evaluates results and stores the resulting information. Therefore, edge computing, where devices gather and process the data, is difficult to apply here, having to focus on cloud or fog computing of the data. In this point, we clarify that the DOLARS architecture described has been deployed in the local network and nodes of UAL, so it is compatible with fog/cloud approaches. Nevertheless, in the next sections we distinguish between fog nodes where the computing processes, such as gateways, are located close to the devices which collect the data, and cloud nodes which configure the approach for on-line AR which are developed in a distributed way without restrictions in terms of physical location for deployment. As there is a huge amount of data (Table 2) to be gathered and processed for determining one activity in a specific range of time (0.5 s), using a single processor could sometimes provoke a *bottleneck* and therefore it would imply that the system would not recognize the activities in real time. Consequently, it is necessary to distribute the data and processing of the window into different nodes or processes by defining a pipeline. For this reason, some tools like cache storage and real time distribution platforms are incorporated into the system.

**Figure 16:**
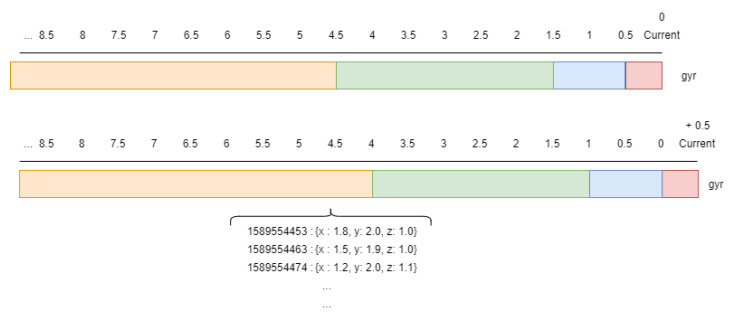
Raw data storage in Redis database.

A computationally expensive part in the system is that it would have to access the database not only to extract the data from the last window but also from the previous windows that had been processed previously. In order to reduce accesses to the database, a cache has been introduced into the system where the data that are read from the database are stored and will be used in future time window calculations. This cache database is integrated by Redis database which let users store data following a key-value pattern that is very useful to this problem. Here, the raw data from each new 0.5-s-window is stored following the pattern “timestamp(key)-data(value)” as shown in Figure 16. In this Figure, the corresponding “gyroscope” sensor windows generated for two consecutive instants are shown, where different colours represent different duration of windows (5.0, 3.0, 1.0 and 0.5 s) indicated in Table 2. Red represents the 0.5 s window that is the only one that contains new data compared to the previous instant. Information on the remaining windows has already be processed in previous instants of time and it is not necessary to access to the database to get this information. To compute the features of each window it is therefore necessary to process all the data defined by them.

Taking into account Table 2, which shows the 13 windows to be considered, initially each time a new window arrives, the database would have to be accessed 13 times. However, considering the new approach, it is only necessary to access the database 4 times, once for each type of sensor defined in Table 2. In these accesses only the information of the smallest window defined for each sensor is read (0.5 s in case of the accelerometer and gyroscope, 1.5 s for location and 5 s for binary sensors). In addition, the amount of time employed on these query operations within a time interval with the external database is widely reduced.

**Figure 17:**
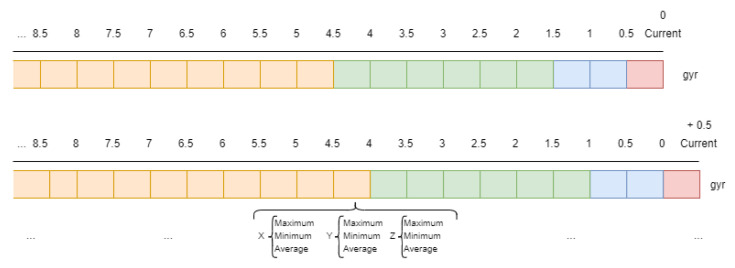
Metrics storage in Redis database.

Computing features from temporal data has been properly optimized in the Redis database thanks to the segmentation of mid-second windows. When new data arrive, the node computes the features of the current window (maximum, minimum and average), storing these metrics rather than raw data, as shown in Figure 17. So when another node computes the features in a given interval, it is only necessary to recover the metrics of mid-second windows.

#### 3.3.4. On-Line Activity Recognition by Means of Real-Time Distributed Services under Publish-Subscribe Model

**Figure 18:**
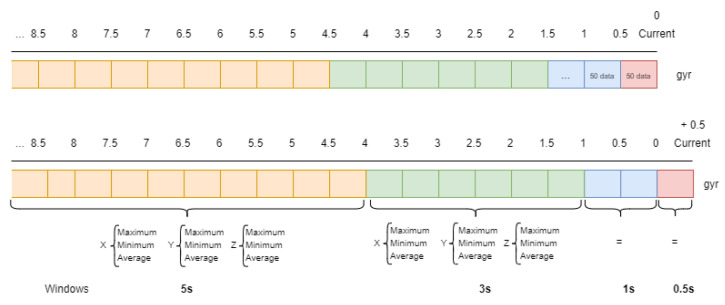
Big windows.

When the computing of features in the cache from the new 0.5-s-window is completed, next, a process has to gather the mid-second windows forming bigger windows as defined in Figure 18. Once these windows are gathered, the overall features have to be calculated to be classified. These operations do not last a fixed amount of time and, in some cases, processing time of features and recognition could be greater than the real-time time-step to develop the AR in real time (0.5 s). This would imply that it would be impossible to achieve a real-time activity recognition.

As mentioned above the MQTT broker is focused on the exchange of messages on many different topics. However, in order to process the massive amount of data in real time, we have selected the Apache Kafka broker, whose focus is storing massive amounts of data on disk, and allowing a distributed consumption in real time under a publish-subscribe paradigm [61]. Apache Kafka has been designed to be deployable as a cluster of multiple nodes, with good scalability properties.

**Figure 19:**
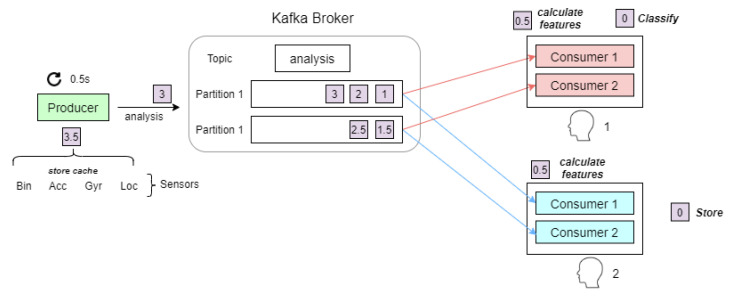
Kafka system.

Therefore the tool of Apache Kafka has been incorporated into our system following the structure shown in Figure 19. A process generates one new datum every 0.5 s which defines the current time, and sends it to the Kafka broker. Inside this broker, different topics can be defined. In the example on Figure 19, the “analysis” topic is used. In addition, each topic has several partitions in order to speed up the processes. In this way, the received messages are distributed among these partitions following a first in, first out system. Finally, we define as many different consumer groups as number of sources producing data the system has. So, in the example, there are two consumer groups running because the system recognizes the activity of two people in the smart home. At each consumer group, there are as many consumer processes as partitions has the topic. Each consumer processes the sensor data which is involved in each received message. Thus, each consumer group receives all the information provided by the producer node.

Once the structure and organization of the nodes system is developed, the operations which take place on the producer node and the consumer node have to be analyzed. On the one hand, the producer node generates new data specifying the current time-stamp each 0.5 s. Once these data are generated, it gathers them from the database, calculates the metrics and stores them in the cache system. After that, it sends data to the Kafka broker and finally the process starts again. About calculating the metrics, it uses the “pandas” library which offers developers some tools to transform the data in an easy way. This method is widely adopted in big data and machine learning problems.

On the other hand, the consumer nodes receive some data referencing the window they have to process. Each consumer node is associated with a partition into the broker that balances data among partitions. Therefore each node gathers data from all the required windows and later calculates their features (maximum, minimum and average). After that, a fusion of these data is done, obtaining a feature vector with 117 features in this system, being followed by a pre-processing process. Finally, a machine learning classifier, which is installed at each node, makes a classification of the input data to recognize an activity.

#### 3.3.5. Classification Models, Pre-Processing of Data and Configuration

As mentioned, some machine learning techniques have been compared in order to determine which one obtains better results. These techniques have been applied in the consumer nodes. In this work, kNN (k-nearest neighbors), RandomForest and SVM (Support vector machine) classification algorithms have been compared in order to select the one having better results.

First, kNN (k-nearest neighbors) is an algorithm which does not generate a model for the classification of the new data, but in order to perform this classification it is based on previously classified instances. To classify a new record, the algorithm calculates the distance between it and each record stored in the algorithm, being classified according to the characteristics of the *k* registers most similar to this one. Second, SVM (Support vector machine) is an algorithm whose main objective is to generate a limitation (hyper plane) among the different classes (activities in this case) in such a way that this limitation is as separate as possible from these classes, thus differentiating the set of data and limiting the “region” corresponding to each class. Therefore, the more separated the hyper plane is from the elements of the classes, the more limited these classes are and the better the classification will be done later. Third, a decision tree is a machine learning algorithm that contains different levels with decisions that lead to a final result. The final result consists of leaf nodes that correspond to the class that is associated with the data that is being classified.

RandomForest is an ensemble of decision trees combined with bagging. When using bagging, different trees see different portions of the data. No one tree sees all the training data. This makes each tree train with different data samples for the same problem. In this way, by combining their results, some errors are compensated with others and we have a better generalising prediction.

Therefore, in this system, kNN, SVM and RandomForest are tuned with different configurations by using the feature “Random Hyperparameter Grid” from scikit-learn. However, in most of cases, the best configuration is the default one offered by the library. Thus, the first model is kNN, which computes the number of neighbours as the square root of the total number of data in the dataset. It uses the “Euclidean” distance as the metric to compute distances between neighbors, and it defines the “distance” magnitude as the weight function, in such a way that close neighbors of a query point have a greater influence than neighbors which are further away. The second resulting model is kNN having 100 number of neighbors, using the “Manhattan” distance and defining a “uniform” weight function where close neighbors have the same influence than the ones which are further away. Third model is SVM, which is tuned by establishing the Radial Basic Function (RBF) as kernel. The fourth model, RandomForest, is configured following the default parameters, as they fit well with the testing dataset. The most important parameters are: number of estimators equal to 100, no max-depth of the trees and the minimum number of samples in the leaf nodes fixed to 2.

In addition, it is interesting to remark that before classifying the data by using some machine learning models, it is necessary to follow a pre-processing step for the data.

Firstly, variable scaling techniques are included. On the one hand, a procedure that normalizes the data to have a distribution with mean equal to 0 and variance 1 is tested. On the other hand, a procedure that scales the data of the characteristics vector to a range of 0-1 is tested [62].

Second, techniques for selecting attributes are included. These range from using an SVM classifier to detect the attributes that are important in the classification to using variance to discard data with little variation.

Finally, the different pre-processing techniques are tested on the current data to see which ones yield better data with the defined data and algorithms.

In this work, several configurations have been adopted in order to evaluate each algorithm. “Default” does not include any pre-processing step; “scaled” includes the data in a range of 0–1; “normalized” implements a standard scaler in the data; “ats_var” includes a feature selection by removing the attributes with low variance; “ats_model” removes the attributes by following their importance in SVM classification and finally, “norm_ats_model” normalizes the data and selects the attributes by following an SVM model.

## 4. Evaluation

In this section, we describe the case studies developed in the work to evaluate the performance of the approach in terms of computing time for on-line AR and accuracy of AR approach based on sequences of daily human activities developed by an inhabitant.

The case studies were developed in UAL, in the new Smart Lab presented in this work in Section 3.1. The sensor devices included in the evaluation are: wearable, ambient and location sensors, which are previously described in Section 3.2.

### 4.1. Experimental Setup

In order to train and evaluate the system, two experiments were carried out. In the first of them, a participant was asked to simulate a daily living experience in the Smart Home during four hours. While this participant was carrying out the activities, he was being observed by a researcher from the observation room, who labelled all the activities performed by the participant. In the second experiment, two participants were asked to simulate a daily living experience in the Smart Home during two hours each. In the second experiment, participants from different sexes and heights were chosen. In both cases, the users were asked to do the activities included in this paper in a natural way and in a logical order. In this way, for example, in most of the cases, the “Brushing teeth” took place after “Eating”. In addition, several activities such as “Smartphone” and “Walk” were trained to take place in all the rooms and “logical” locations in the Smart Home. In most cases, the activities that were recorded had the same duration as they have in a real environment. However, some activities such as “Sleep” or “Shower” were not performed completely because of their duration. Also, in the second experiment, participants performed the activities with the normal variations that two different users can have, for instance, brushing teeth. Therefore, as a result of the training process, two datasets were generated, the first of them having 8940 instances and second one having 7857.

**Figure 20:**
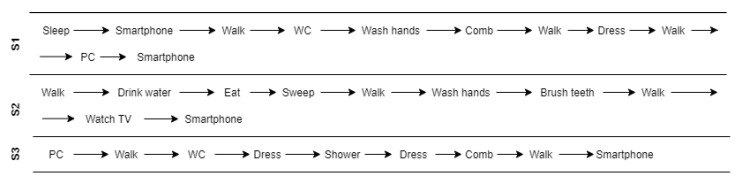
Fixed-Sequences of activities.

After recording the activities and generating the respective models by following the configuration shown in Table 3 the models were evaluated in a real time environment. In the first experiment, the same participant performed three sequences of activities. In the second one, another participant which did not train the model was asked to perform the three sequences of activities, following the leave-one-subject-out approach. These sequences are shown in Figure 20. As can be appreciated, they are a common sequence of activities which a lot of inhabitants can do in their daily life.

In this system, the processing of the data takes place in the cloud. Specifically, it is deployed in several virtual machines deployed using Open Stack, an open source cloud computing infrastructure software project. Thanks to this platform, UAL provides the students the ability to use a private cloud. Thus, in this project, several processes which are always running, take place in a machine with the following features: Ubuntu 18.04 LTS as OS, 8GB RAM, 4V CPU and 80 GB of HDD disk. It is enough to execute the scripts that are part of the activity recognition system. In this way, once the results and conclusions are obtained, this data is sent to the public cloud in order to see the results everywhere.

### 4.2. Algorithm Evaluation

In order to train and evaluate the different algorithms and configurations, several cross-validations have been applied to the dataset. The first one includes 70% of samples from dataset to train the model and 30% to evaluate. The second one has 80% of samples to train the model and 20% to evaluate. Table 3 show the algorithms with the aforementioned pre-procesing configurations that have been tested and compared by using some cross-validation processes in experiment 1. In Accuracy1 the accuracy value from the first cross validation process (70-30) is shown and Accuracy2 shows the results from the second cross-validation process (80-20). Average Accuracy shows the average of these values.

As can be seen, RandomForest classifier provides better results as it obtains 99% accuracy. The RandomForest classifier (consisting of 100 decision trees in this case) allows a better generalization of the activities included in the dataset and more accurately predicts the new activities. In addition, scaling the attributes from the feature vector is the best configuration (having 99.31% accuracy in experiment 1 and 99.74% in experiment 2), not being necessary to remove some attributes ( 117 attributes were used) and making the pre-processing process less computationally expensive.

However, there are several algorithms and configurations which also lead to good results in terms of activity recognition. First, there are several kNN configurations, one of them establishes k = sqrt(total samples in dataset) and the other one *k* = 10. In both of them, the “scaled” configuration has a better degree of accuracy than the “default” one. This is because in the feature vector, there is a fusion of all sensors data, which have different ranges and values. As kNN measures the “distance” from each value to all the other values in the dataset, the features having more units have more importance. By scaling all these attributes to the range of 0–1, all the attributes have the same importance and therefore, so the kNN works better. In the same way, when normalizing the data, this algorithm works well. Nevertheless, in this case, SVM and RandomForest have better results.

Contrarily, the SVM can reach 99.27% accuracy in experiment 1. The configuration which allows this is normalizing the data and selecting the attributes in them. Thus, SVM is considered to work well when normalizing the data, as for some cases, it calculates the different hyper planes by using the euclidean distance, which is sensible to different scales between features. In this case, it is the best configuration of the SVM algorithm.

### 4.3. Activity Recognition Performance

The activity recognition algorithm includes a RandomForest classifier, which uses scaled data in order to make its classifications.

Once the models were trained with 80% of the samples in the dataset, they were evaluated with the 20% remaining samples. In Table 4 and Table 5, the values of precision, recall and f1-score of all the activities in the dataset for both experiments are shown. First, the precision is the ability of the classifier not to label as positive a sample that is negative. Second, the recall is the success of the classifier at finding all the positive samples. Third, the F1 score can be interpreted as a weighted average of the precision and recall, where an F1 score reaches its best value at 1 and worst score at 0.

In general, all the activities achieve good results in terms of F1-score (8 out of 14 activities have the value 1.0 in experiment 1 and 12 out 14 in experiment 2). Indeed, all of them have a value equal or higher than 0.97.

In particular, as an example of not achieving 100% success in experiment 1 is the case where “Dress” is classified incorrectly, making this activity have a 0.97 in precision value. Similarly, in experiment 2, the activity “Comb” has the lowest value, 0.98.

Moreover, in the first experiment, activities like “Wash hands” and “Comb” have the lowest values in terms of recall, meaning that these activities are not always recognized correctly. In the second experiment, the activity with the lowest value is “Brush teeth”.

**Figure 21:**
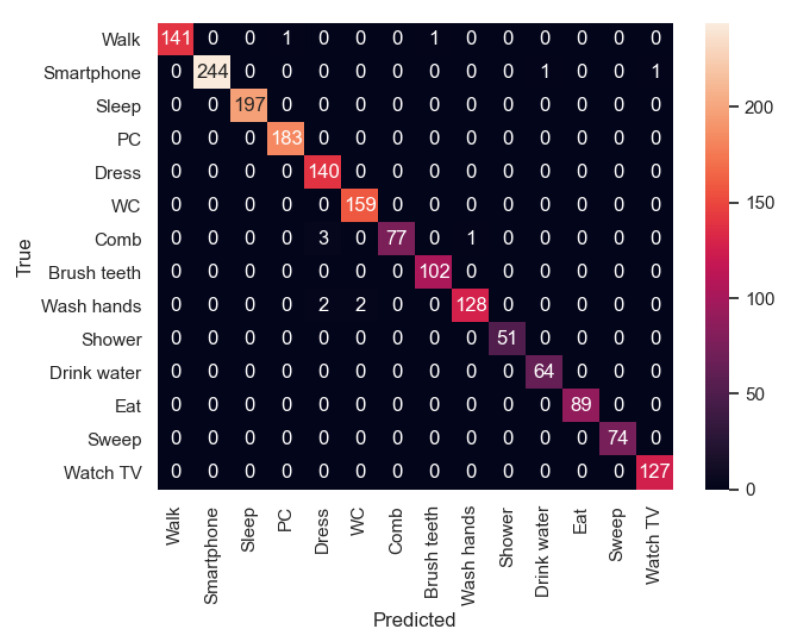
Confusion matrix of the experiment 1.

**Figure 22:**
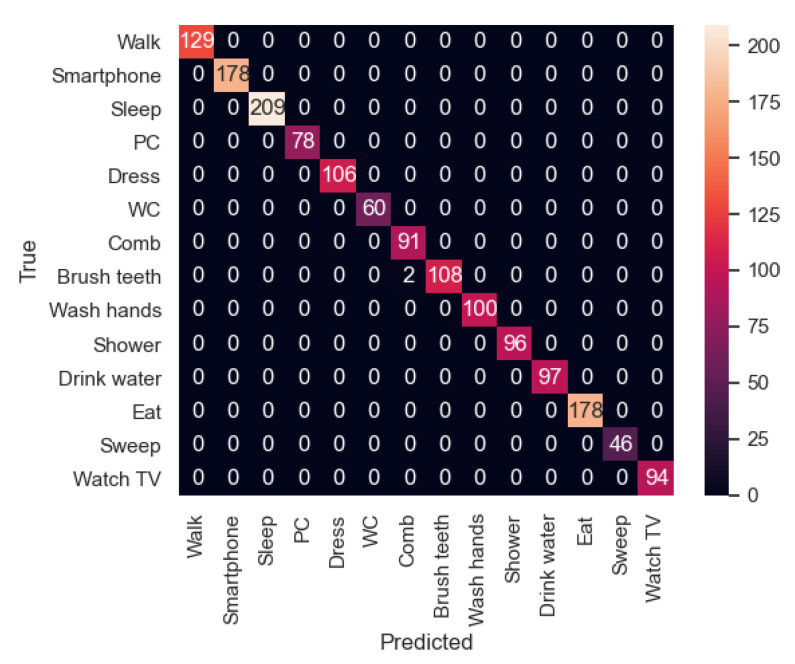
Confusion matrix of the experiment 2.

In Figure 21 and Figure 22 the confusion matrices of the activity recognition system resulting from both experiments are shown. Here, we can appreciate that almost all the classifications are correct. However, experiment 2, which was trained with two participants instead of a single one, has better results due to its generalization of the activities.

First, as mentioned before, in the first experiment, the activity “Dress” has a 0.97 value in terms of precision as it has been classified incorrectly 5 times (3 times with “Comb” activity and 2 times with “Wash hands”). Among these activities, there is some confusion because the movements of the smartwatch are so similar, the place where they are carried out is in some cases the same and the binary sensors have similar values in the bathroom. In the case of the second experiment, a similar behaviour can be appreciated in the “Brush Teeth” activity, which is classified twice as “Comb”. This could be improved by adding some inertial sensors on the relevant daily-use objects like a toothbrush or a comb.

In general, activities which are carried out in the bathroom are more difficult to differentiate from each other, because in some cases, the location sensor is placed in a static way while the user is having a shower or dressing.

Moreover, once the RandomForest was chosen and a scaling data pre-processing was also applied, a participant was asked to do three sequences of activities in the Smart Home in order to evaluate the activity recognition algorithm in real time. In the first experiment, the participant was the same that trained the model. However, in the second experiment, the participant was new.

**Figure 23:**
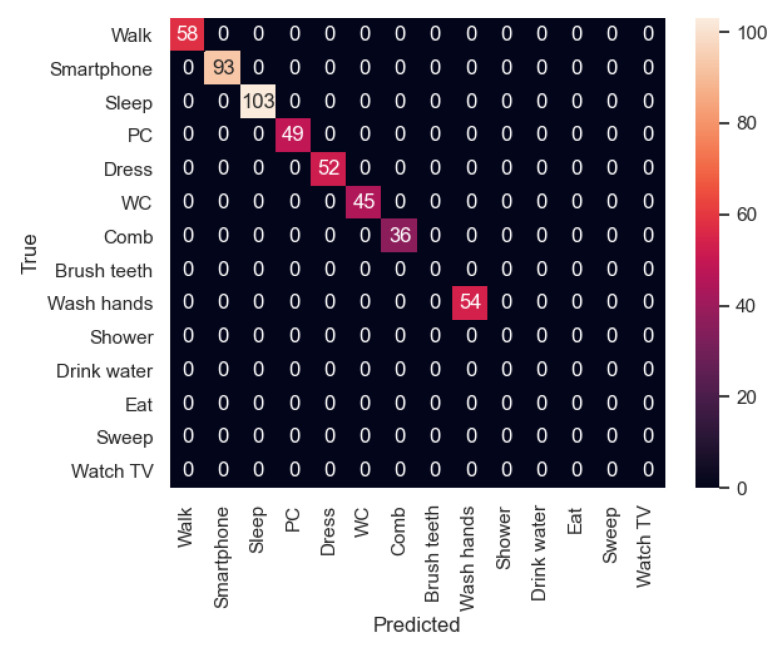
Sequence 1-Experiment 1-Confusion Matrix.

**Figure 24:**
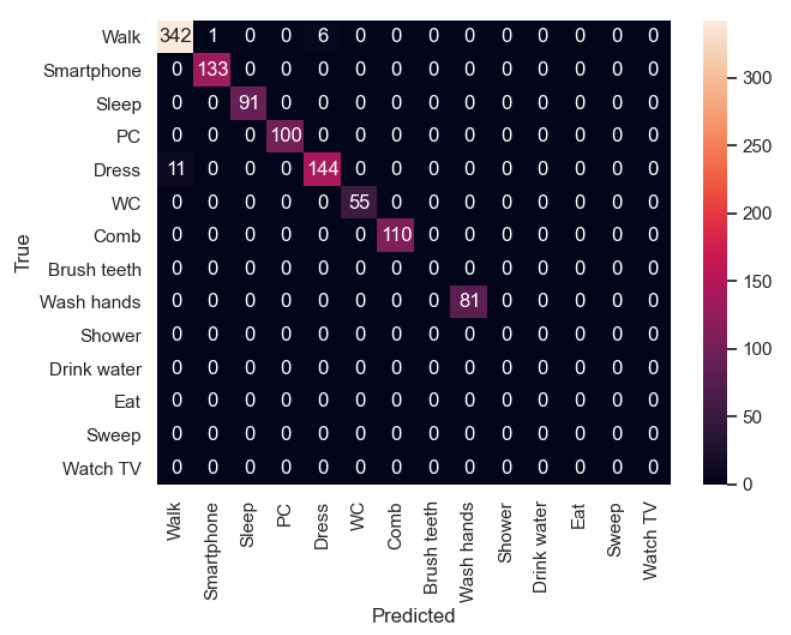
Sequence 1-Experiment 2-Confusion Matrix.

In Figure 23, the confusion matrix of the first sequence in the first experiment is shown. In this case, the participant’s activities were correctly recognized. However, in the second experiment (Figure 24), the new participant performed the activities in a slightly different way from the other participants, and therefore there are some activities such as “Dress” that were recognized as “Walk” in some cases. In addition, “Walk” was not always recognized, being classified 6 times as “Dress”. In this sequence, we can appreciate that each user dresses in a different way, indicating that the model has to be trained with more participants in order to be better generalizable.

**Figure 25:**
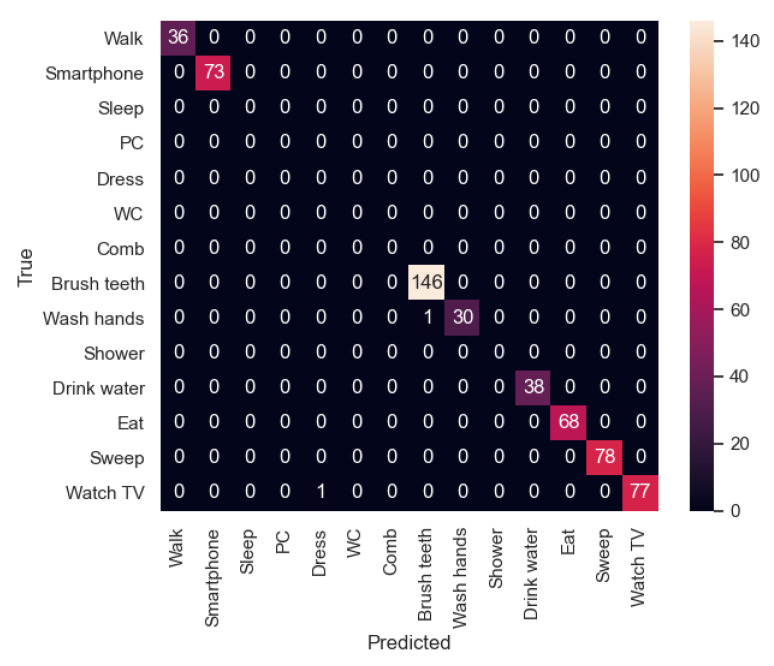
Sequence 2-Experiment 1-Confusion Matrix.

**Figure 26:**
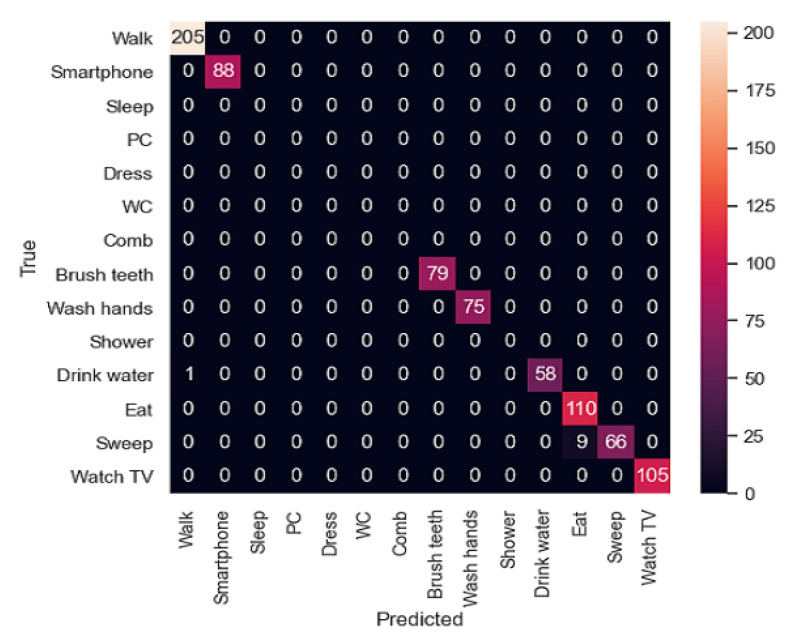
Sequence 2-Experiment 2-Confusion Matrix.

In the second sequence, in both experiments, most of the activities were well recognized, as shown in Figure 25 and Figure 26.

**Figure 27:**
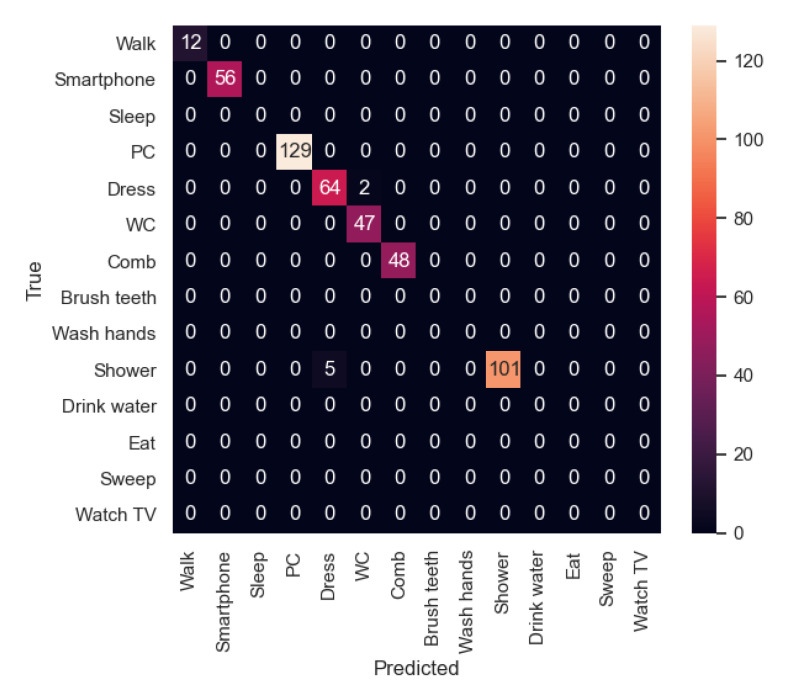
Sequence 3-Experiment 1-Confusion Matrix.

**Figure 28:**
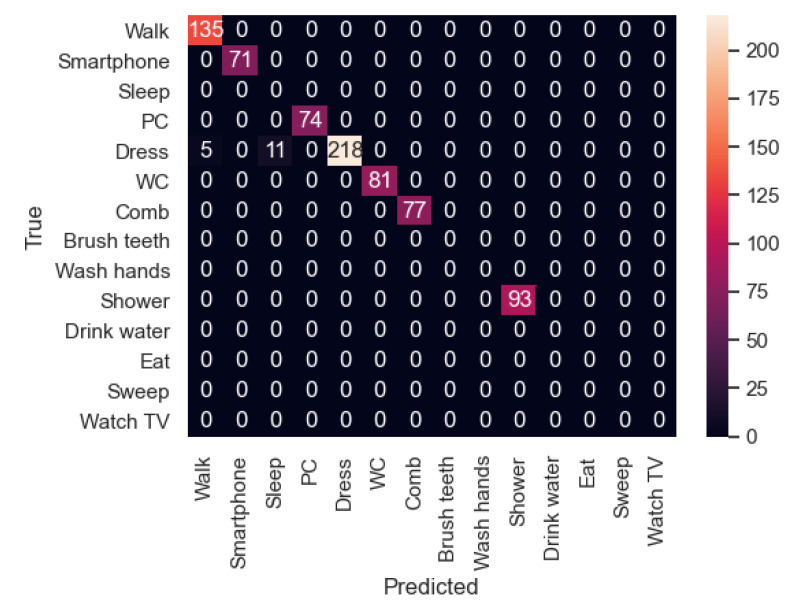
Sequence 3-Experiment 2-Confusion Matrix.

In the third sequence, the first experiment (Figure 27), sometimes classified the “Shower” activity as “Dress”. As it was explained before, there could be some bathroom activities which tend to be detected as “Dress”. This also can be possible if the motion sensor which is located in the bath detects a user who is being undressed before having a shower. However, in the second experiment (Figure 28), the “Dress” activity is sometimes classified as “Sleep” and “Walk”. This is because the “Dress” activity is trained to be done in the bedroom and the bathroom. In future work, this activity has to be better trained, as mentioned before.

To summarize, the recognition of activities in real sequences shows that the algorithm correctly classifies new data generated in a real environment. In the first experiment, the model was better adapted to the participant because it was trained by using data generated by the same person. The downside of this is that the model could perform worse when a new participant is included in the system. The addition of a different participant in the evaluation of the system was observed in experiment 2, where the model was trained with activities of two participants and evaluated by a different one. With regard to the results showed before, the model decreases its accuracy in evaluation but it maintains performance in terms of activity recognition. Thus, training the system following the steps indicated in this paper allows the system to achieve excellent results in terms of natural conditions, though it requires a previous training stage. In this sense, the advantages of using this approach is the customization and good performance in the learning. However, the disadvantage is that it requires time and training data before the activity recognition stage.

### 4.4. Distributed Architecture Performance

**Figure 29:**
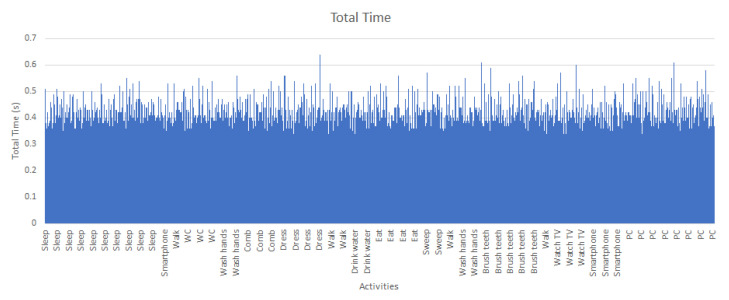
Total processing time of a sequence of activities.

After running all experiments it is interesting to remark that the average window processing time in recognizing an activity remains the same regardless the current activity. In Figure 29 the processing time of a sequence of activities carried out by a user is shown. It is appreciated that only a few windows have a duration greater than a half-second. In addition, the activities have a similar behaviour, independent of the sensor data which each one involves.

**Figure 30:**
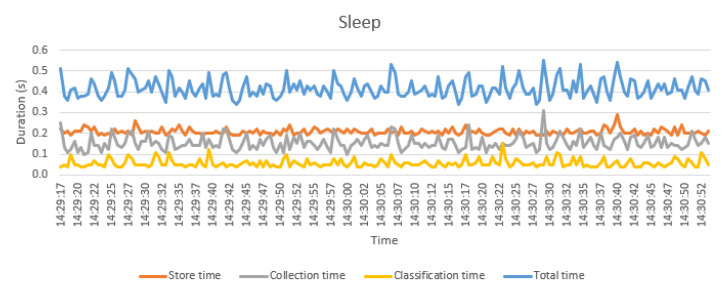
Processing time for sleep activity.

In Figure 30, the different time measurements for the activity “Sleep” are displayed though almost the same time distribution is obtained for all the activities. The average processing time is about 0.42 s and includes several time measurements: storing, collection and evaluation time. First, average storing time, which includes the query of the data from the sensors, the calculation of the metrics and the storage of the metrics in the cache, lasts 0.15 s. Second, the collection time, which includes the building of all the windows, the gathering of data which corresponds to each window, the obtaining of their metrics and the providing the feature vector, requires 0.21 s. Finally, the evaluation time, which includes the pre-processing of the feature vector and its classification, lasts only 0.05 s.

It can be analyzed that the storing time is the more computational cost procedure, while the classification requires less time. However, it is remarkable that storing and evaluation time do not depend on the window sizes defined in the system while the collection time increases as the window sizes do.

Considering that for the proposed system the average processing time to recognize a 0.5-s-window size is 0.42 s it could be deduced that a single processor is enough to recognize the activities in real time. However, the processing time is not constant and on some occasions lasts more than 0.5 s and therefore in those cases it would be impossible to start processing the new arriving window and a delay in the system will be produced. For this reason, a distributed architecture by using Kafka has been included in the system. In this architecture, a node gathers and stores the data and other nodes get these data, process them and classify some activities. This distributed system will define as many nodes as necessary to be able to start processing each new window at the same time it arrives.

Table 6 shows some time data when a different number of consumers or nodes are defined in the system, that is, the average total time of processing a mid-second window, the corresponding standard deviation and the average waiting time where the consumers are idle and waiting for a window to process. As can be appreciated, the processing time is almost the same in all the configurations. This means that the introduction of the distributed system does not produce an overload on the system and the windows are processed in the same computation time (about 0.42 s) independent of the number of processors or nodes.

Regarding the waiting time, it can be observed that it increases as the number of consumers does. In the case of a single processor, the average waiting time is 0.07 s. It happens because the system with a single processor is able to classify a 0.5-second-window in an average time of 0.42 s and the process in average waits 0.07 s until a new window arrives. Of course, as has been explained before, if some windows require more than 0.5 s to be processed, there could be a bottleneck in the system that could cause the system to stop classifying activities in real time. However, using two consumers, the bottleneck is always avoided. In this case the average waiting time is smaller than 0.5 s. This means that in some cases the two processors or consumers are working simultaneously, which justifies the need for using more than a single processor. In the case of 3 and 4 consumers the average waiting time increases as there are more processes than required for the case study. Consequently it can be concluded that for the case of a single user and the window sizes defined in Table 2 only two consumers are required to allow the classification in real time. In spite of the fact that for this case study no more than two consumers are necessary, when the window processing time is greater than 0.5 s more consumers may be demanded. In order to analyze how the processing time varies with the window sizes, a new experiment has been carried out where the two new window size configurations have been defined. So, in Table 7 the configuration 0 coincides with the one that has been considered throughout the paper, and the two new configurations have incrementally larger window sizes.

For these three configurations, the processing time when classifying activities have been computed and average results are provided in Table 8. It can be seen that storing and evaluation time only slightly increases with the configuration of the window sizes. However, collection time strongly depends on this sizes and therefore the total processing time also increases. Therefore for configuration 1 the processing time (0.83 s) is almost double than for configuration 0. Analogously the processing time for configuration 2 (1.34 s) is more than three times that of configuration 0. Accordingly, for configurations 1 and 2 more than 0.5 s are required to process a window. That means that a single processor cannot classify in real time as every 0.5 s a new window arrives to the system to be processed and classified. For configuration 1 at least two consumers will be required or even three if the time processing peaks are very frequent. In the same way, for configuration 2 at least three consumers will be demanded in order to allow the system to classify in real time.

In addition it is noteworthy that all previous experiments have been carried out considering a single user in the smart home. However when there is more than a single user in the Smart Home, more consumers or processors can be straightforwardly included to keep the real time system conditions in processing multi-occupancy thanks to the scalable architecture here proposed.

## 5. Conclusions and Ongoing Works

In this paper DOLARS, a Distributed On-Line Activity Recognition System, is presented. In this platform data from heterogeneous sensors are evaluated in real time, including binary and new generation of wearable and location sensors deployed in the new Smart Lab of UAL in Spain.

The new Smart Lab has been introduced and the different rooms and sensors and actuators already installed are mentioned. Later the infrastructure of the IoT system has been described and the working frequencies of each sensor have been indicated. Taking into account the difference in nature and rate of sensor, a set of different time windows has been determined in order to create a set of sliding windows that fixes a 0.5-s current window (time-step). Every time a new current 0.5-s-window arrives, the system has to access the database not only to extract the data from the last window but also from the previous windows that had been processed before.

In order to be able to process all the information in real time some improvements have been included into the system. First, a cache has been introduced to the system where the data that are read from the database are stored and will be used in future time window calculations. This cache database is integrated by Redis database which lets developers store data following a key-value pattern that is very useful to aggregate sensor data in real time. Second, considering the large amount of data at each temporal window, the information stored in Redis database has been reduced to compute the metrics of the feature vector. So, when another node computes the features in a given interval, it is only necessary to recover the metrics of mid-second windows. Third, the Apache Kafka broker has been deployed because its focus is storing massive amounts of data on the disk and allowing a distributed consumption in real time under the publish-subscribe paradigm.

Additionally, with the aim of selecting an efficient and effective machine learning algorithm to recognize the different user activities, three techniques (KNN, RandomForest and SVM) have been compared using several pre-processing methods. After analyzing the results, RandomForest with scaled data has been selected to be integrated into the DOLARS platform.

Results for the case studies are shown. The first was trained and evaluated with the same participant, with 99% accuracy. The second, which was trained with two participants and evaluated with a different one, also has 99% of accuracy, with good generalizability of the activities included in the activity recognition system. In addition, the system is able to process the data in real time using two consumers or processors from the distributed system. If the number of users increases, the system is still able to recognize in real time by using more consumers or processors. In the same way, when the past-window-sizes increase, though the processing time is also incremented, the system is able to process data in real time by increasing the number of consumers.

As ongoing work, new strategies to increase the performance of the system will be implemented in order to decrease the processing time, such as implementing a hierarchical data cache where metrics at different granularity could be collected, as is the case in data warehouse systems.

## Figures and Tables

**Table 1:** Total price of the system.

Type of Sensor	Number of Units	Unit/Price	Total Price
Fibaro Motion Sensor	3	49	147
Fibaro Door/Window Sensor 2	6	38	228
Fibaro Door/Window Sensor	3	38	114
Arun PM3	3	12	26
Raspberry Pi	2	60	120
Razberry Module	1	59	59
Polar M600	1	250	250
Decawave MDEK1001 (12 tags)	1	200	200
Total	31	-	1144

**Table 2:** Sliding window sizes and the total quantity of data gathered by each type of sensor. S = Duration in seconds, T = Total generated data in the window.

	Current Window		Past Window-1		Past Window-2		Past Window-3	
**Sensor Type**	**S**	**T**	**S**	**T**	**S**	**T**	**S**	**T**
Binary	5	10	30	60	-	*-*	-	*-*
Location	1.5	1–2	6	6	10	10	-	*-*
Accelerometer	0.5	50	1	100	3	50	5	500
Gyroscope	0.5	50	1	100	3	50	5	500

**Table 3:** Accuracy from different machine learning algorithms and configurations in the experiment 1.

Algorithm	Configuration	Accuracy1	Accuracy2	Average Accuracy
kNN_sqrt	default	83.59	83.89	83.74
kNN_sqrt	scaled	91.69	91.78	91.73
kNN_sqrt	normalized	89.86	89.77	89.81
kNN_sqrt	ats_var	83.59	83.89	83.74
kNN_sqrt	ats_model	83.26	83.84	83.55
kNN_sqrt	norm_ats_model	90.08	90.16	90.12
kNN_10	def	93.7	94.24	93.97
kNN_10	scaled	97.76	98.27	98.02
kNN_10	normalized	96.68	96.59	96.64
kNN_10	ats_var	93.7	94.24	93.97
kNN_10	ats_model	93.77	94.24	94.0
kNN_10	norm_ats_model	96.79	96.81	96.8
RandomForest	def	98.99	99.22	99.1
RandomForest	scaled	99.25	99.38	99.31
RandomForest	normalized	99.11	99.27	99.19
RandomForest	ats_var	99.14	99.38	99.26
RandomForest	ats_model	99.33	99.16	99.25
RandomForest	norm_ats_model	99.18	99.33	99.25
SVM	def	94.59	94.3	94.44
SVM	scaled	97.54	97.43	97.49
SVM	normalized	98.84	99.11	98.97
SVM	ats_var	94.59	94.3	94.44
SVM	ats_model	94.18	94.02	94.1
SVM	norm_ats_model	98.96	99.27	99.11

**Table 4:** Algorithm results in experiment 1.

Activity	Precision	Recall	f1-Score
Walk	1.0	0.99	0.99
Smartphone	1.0	0.99	1.0
Sleep	1.0	1.0	1.0
PC	0.99	1.0	1.0
Dress	0.97	1.0	0.98
WC	0.99	1.0	0.99
Comb	1.0	0.95	0.97
Brush teeth	0.99	1.0	1.0
Wash hands	0.99	0.97	0.98
Shower	1.0	1.0	1.0
Drink water	0.98	1.0	0.99
Eat	1.0	1.0	1.0
Sweep	1.0	1.0	1.0
Watch TV	0.99	1.0	1.0

**Table 5:** Algorithm results in experiment 2.

Activity	Precision	Recall	f1-Score
Walk	1.0	1.0	1.0
Smartphone	1.0	1.0	1.0
Sleep	1.0	1.0	1.0
PC	1.0	1.0	1.0
Dress	1.0	1.0	1.0
WC	1.0	1.0	1.0
Comb	0.98	1.0	0.99
Brush teeth	1.0	0.98	0.99
Wash hands	1.0	1.0	1.0
Shower	1.0	1.0	1.0
Drink water	1.0	1.0	1.0
Eat	1.0	1.0	1.0
Sweep	1.0	1.0	1.0
Watch TV	1.0	1.0	1.0

**Table 6:** Comparison of different consumer performance.

Number of Consumers	Average (Time)	Standard Deviation (Time)	Waiting Time
1	0.42	0.047	0.07
2	0.43	0.051	0.47
3	0.43	0.049	0.84
4	0.43	0.048	1.27

**Table 7:** Window sizes from the different configurations.

Sensors	Configuration 0	Configuration 1	Configuration 2
Accelerometer	0.5-1-3-5	0.5-1-3-5-8-13	0.5-1-3-5-8-13-21-34
Gyroscope	0.5-1-3-5	0.5-1-3-5-8-13	0.5-1-3-5-8-13-21-34
Location	1.5-6-10	1.5-6-10-13-21	1.5-6-10-13-21-34-55
Binary	5-30	5-30-144	5-30-144-233

**Table 8:** Processing time (in seconds) for different window size configurations.

Time (Seconds)	Configuration 0	Configuration 1	Configuration 2
Storing time	0.20	0.21	0.24
Collection time	0.16	0.51	0.94
Evaluation time	0.06	0.10	0.15
Total processing time	0.42	0.83	1.34

## Data Availability

The data presented in this study are openly available in FigShare at doi 10.6084/m9.figshare.13530671 and 10.6084/m9.figshare.13530596.
